# Epidemiological and clinical profile of paediatric malaria: a cross sectional study performed on febrile children in five epidemiological strata of malaria in Cameroon

**DOI:** 10.1186/s12879-017-2587-2

**Published:** 2017-07-17

**Authors:** Tebit Emmanuel Kwenti, Tayong Dizzle Bita Kwenti, Andreas Latz, Longdoh Anna Njunda, Theresa Nkuo-Akenji

**Affiliations:** 1 Department of Medical Laboratory Sciences, University of Buea, P.B, 63 Buea, Cameroon; 2 Department of Microbiology and Parasitology, University of Buea, P.B, 63 Buea, Cameroon; 3 Diagnostic laboratory, Regional Hospital of Buea, P.B, 32 Buea, Cameroon; 4 Research and Development Department, NovaTec Immundiagnostica GmbH, Dietzenbach, Germany

**Keywords:** Paediatric malaria, Uncomplicated malaria, Severe malaria, Prevalence, Epidemiological strata, Cameroon

## Abstract

**Background:**

In the wake of a decline in global malaria, it is imperative to describe the epidemiology of malaria in a country to inform control policies. The purpose of this study was to describe the epidemiological and clinical profile of paediatric malaria in five epidemiological strata of malaria in Cameroon including: the Sudano-sahelian (SS) strata, the High inland plateau (HIP) strata, the South Cameroonian Equatorial forest (SCEF) strata, the High western plateau (HWP) strata, and the Coastal (C) strata.

**Methods:**

This study involved 1609 febrile children (≤15 years) recruited using reference hospitals in the five epidemiological strata. Baseline characteristics were determined; blood glucose level was measured by a glucometer, malaria parasitaemia was assessed by Giemsa microscopy, and complete blood count was performed using an automated hematology analyser. Severe malaria was assessed and categorized based on WHO criteria.

**Results:**

An overall prevalence of 15.0% (95% CI: 13.3–16.9) for malaria was observed in this study. Malaria prevalence was significantly higher in children between 60 and 119 months (*p* < 0.001) and in Limbe (C strata) (*p* < 0.001). The overall rate of severe malaria (SM) attack in this study was 29.3%; SM was significantly higher in children below 60 months (*p* < 0.046). Although not significant, the rate of SM was highest in Maroua (SS strata) and lowest in Limbe in the C strata. The main clinical phenotypes of SM were hyperparasitaemia, severe malaria anaemia and impaired consciousness. The majority (73.2%) of SM cases were in group 1 of the WHO classification of severe malaria (i.e. the most severe form). The malaria case-fatality rate was 5.8%; this was higher in Ngaoundere (HIP strata) (*p* = 0.034).

**Conclusion:**

In this study, malaria prevalence decreased steadily northward, from the C strata in the South to the SS strata in the North of Cameroon, meanwhile the mortality rate associated with malaria increased in the same direction. On the contrary, the rate of severe malaria attack was similar across the different epidemiological strata. Immunoepidemiological studies will be required to shed more light on the observed trends.

## Background

Malaria is a disease associated with great morbidity and mortality especially in children in sub-Saharan Africa (SSA). According to the WHO, there were 214 million cases and 438,000 deaths attributed to malaria in 2015 [1]. A vast majority of cases and deaths occurred in children in SSA [1]. A declining trend has been observed in the global incidence of malaria in recent years; compared to the year 2000 (where global incidence stood at 262 million cases and 839,000 deaths), the incidence and number of deaths due to malaria have decreased by 18% and 48% respectively [1]. A similar trend has been observed in paediatric malaria where the incidence decreased from 33% in 2000 to 16% in 2015 [1]. Most of the reduction in malaria incidence and deaths was in Africa chiefly in Central Africa [2]. Although malaria incidence is decreasing, there has hardly been a reduction in the role of malaria as a major cause of death in children particularly in SSA, claiming the life of a child every 2 min [1].

In malaria endemic areas, the rate of severe malaria attack varies considerably from as low as 6.4% to as high as 74.7% [3–11]. In children, the clinical spectrum of malaria usually ranges from asymptomatic carriage of malaria parasites to a febrile disease that may evolve into a severe, life-threatening illness [12]. The mortality resulting from malaria is largely associated with the parasite’s ability to induce complications presenting as cerebral malaria, severe anaemia, and respiratory distress (also known as acidotic breathing). In addition other severe malaria manifestations commonly observed at enrollment include: hyperlactataemia, multiple or prolonged convulsions, hyperparasitaemia, circulatory collapse, hypoglycaemia, prostration, jaundice, persistent vomiting and intravascular haemolysis [5, 13, 14].

Cameroon, a malaria endemic country in Central Africa has witnessed a decline in the incidence of malaria, largely attributed to the relentless effort by Cameroon’s government to ensure that every household in the nation is entitled to insecticide treated bed nets (ITNs) [15, 16]. Although malaria has lost its crown as the number one cause of mortality in Cameroon [17], it is still a major cause of morbidity and mortality especially in children. In Cameroon, malaria accounts for 48% of all hospital admissions, 30% of morbidity and 67% of childhood mortality per year [18, 19]. Records show that the entire Cameroon’s population of over 22 million is at risk of malaria infection [17]. Moreover, severe malaria cases (including cerebral and severe anaemia, which are known to be the two major contributors to overall malaria mortality, are frequent in Cameroon [20–23].

The epidemiology of malaria in Cameroon has been described as unique, having all the different epidemiological strata present in all of Africa [24, 25]. Six epidemiological strata have been identified and mapped in Cameroon namely: the Sudano-sahelian strata, High inland plateau strata, Savannah-forest transmission strata, South Cameroon Equatorial forest strata, High western plateau altitude strata, and the Coastal strata [25]. These epidemiologic strata differ in terms of their geographical and ecological characteristics, transmission pattern and endemicity level, and in terms of the main vectors transmitting malaria parasites [25]. Due to these differences, it is likely that exposure and the risk of development of severe malaria among children (who present the most vulnerable group) will vary considerably across the different epidemiologic strata of malaria, which may have particular implications in the control of malaria in the country.

As Cameroon pursues its ambitious goal to reduce malaria-associated morbidity and mortality and eventually eliminate malaria from the country, there is an urgent need of empirical studies to establish an evidence-based distribution of malaria and its clinical features in the population living in the different epidemiological strata that supposedly characterize malaria transmission pattern in the country. Although a few studies aimed at describing the clinical and epidemiological profile of paediatric malaria have been performed in Cameroon, none of these studies has actually taken into consideration populations living in the different epidemiological strata. In view of this, we carried out this survey to describe the epidemiology of malaria and its clinical features in children residing in five epidemiological strata, so as to inform control policies in the country.

## Methods

### Study area

**Fig. 1:**
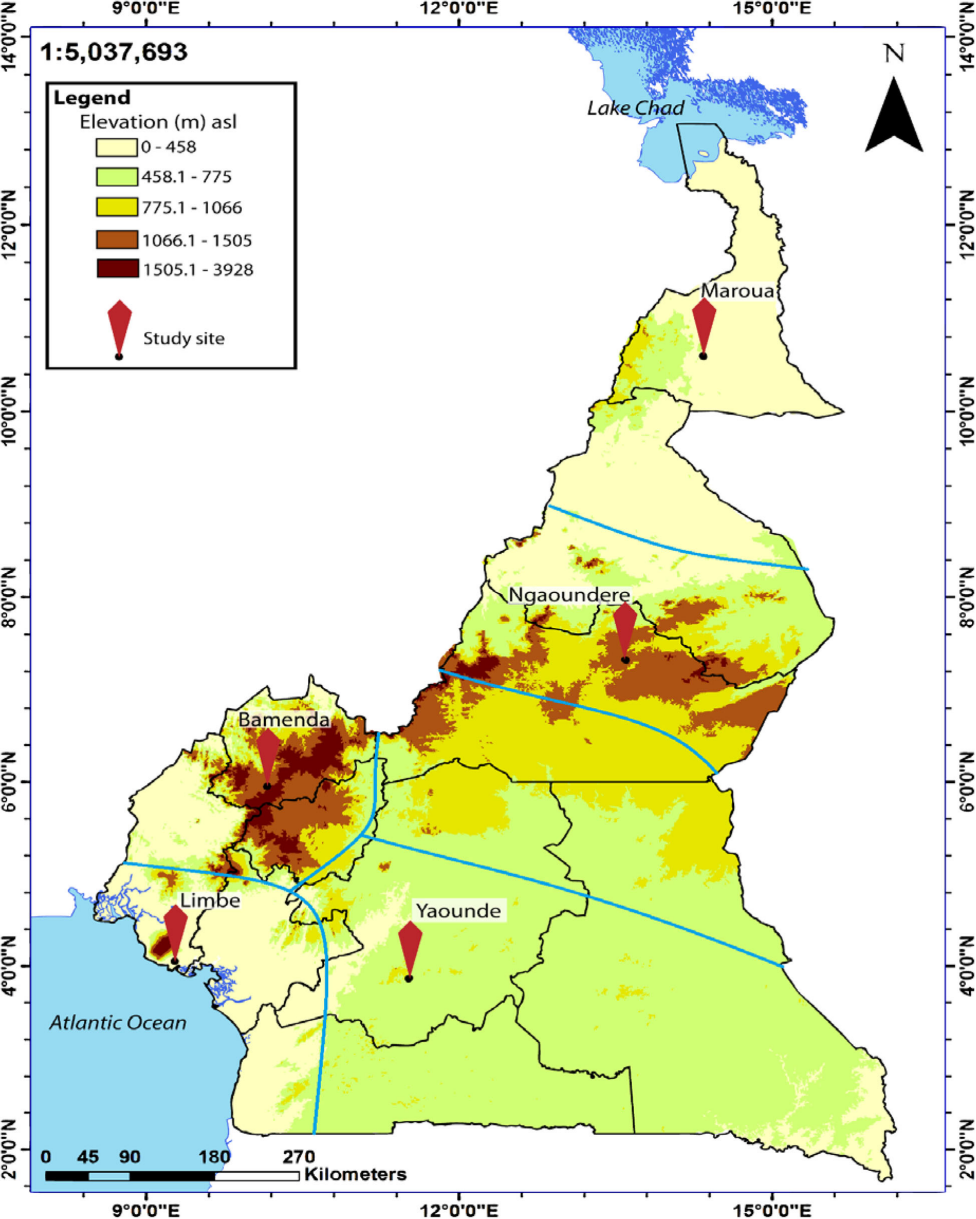
Map depicting the study sites selected. Five epidemiological strata are delineated

Five out of the 6 epidemiological strata of malaria in Cameroon were randomly selected for this study. Five study sites, each representing the epidemiological strata were further selected and included: Maroua in the Sudano-sahelian (SS) strata, Ngaoundere in the High inland plateau (HIP) strata, Yaounde in the South Cameroonian Equatorial forest (SCEF) strata, Bamenda in the High western plateau (HWP) strata, and Limbe in the Coastal (C) strata (Fig. 1). The characteristics of the different strata have previously been described [25].

### Study design and duration

This study was a cross sectional study involving children who came to consult in the OPD/Emergency units of the Regional Hospitals in the different study sites. Data was collected between May and November 2015 (to coincide with the rainy season during which transmission is highest), simultaneously in the different study sites.

### Sample size estimation

The sample size was estimated using the formula for sample size calculation described by Swinscow [26] as follows;

$$ \mathrm{n}=\frac{Z^2 x\  p\left(1- p\right)}{e^2} $$

$$ \mathrm{Z}=1.96 $$

p = prevalence of malaria in Cameroon = 29% [27].

e = error rate = 0.05

$$ \mathrm{n}=\frac{1.96^2 x\ 0.29\left(1-0.29\right)}{0.05^2}=316.4\approx 317 $$

Thus, we recruited 317 participants per study site giving an overall total of 1585 for the 5 sites.

### Participants and sampling

Febrile children (≤15 years) who came to consult in the outpatient department or emergency unit of the District and Regional Hospitals in the different study sites were considered. Convenient and consecutive sampling was used to select participants once they agreed to participate. The vital signs of the patients were taken and they were examined by the consulting physicians.

Excluded from the study were patients with a history of anti-malarial treatment within one week prior to enrollment. Patients were also excluded from the study based on evidence of other infectious disease, such as typhoid, gastroenteritis, meningitis, malnutrition, upper respiratory tract infections or any other identified cause of anaemia other than malaria.

### Laboratory analyses

#### Specimen collection

About four (4) ml of blood was collected from consented participants into EDTA anticoagulated test tubes using aseptic techniques. Blood in the EDTA anticoagulated test tubes were used for the performance of the complete blood count (CBC) and preparation of thick and thin blood films for malaria screening.

#### Measurement of blood glucose level

Blood glucose level was measured by a glucometer, On.Call®Plus Blood Glucose Meter (ACON Laboratories, Inc., USA) using capillary blood from a finger prick.

#### Performance of complete blood count (CBC)

CBC was performed using the Mindray® Auto haematology analyzer (BC-2800, Shenzhen Mindray Bio-Medical Electronics Co., Ltd). The haemoglobin concentration (Hb) and white blood cell counts were obtained from the CBC results.

#### Detection of malaria parasite

The prepared blood films were air-dried and stained with 10% Giemsa (1 in 20 dilutions) for 25-30 min [28]. The blood films were read by two expert microscopists who were blinded from the results of the other. In the case of any discrepancy with the results obtained by the two microscopists, a third was brought in and the results he gave was considered as final. At least 200 fields were screened for malaria parasite using the 100X (oil immersion) objective and where parasites were seen, they were counted until 500WBC were reached. The slides were only declared negative after counting to 2500WBC. Malaria parasite density was estimated by dividing the parasites counted by 500 WBC and then multiplied by the actual WBC count of the participant to give numbers in parasite per μl [29].

#### Categorization of malaria into uncomplicated and severe forms

Severe malaria (SM) defined by the presence of asexual parasitaemia in addition to at least one of the following WHO [30] criteria; 1) severe anemia (Hb < 5 g/dl) with no history of severe bleeding; 2) prostration, defined as the inability to sit or eat in children that were able to do so; 3) respiratory distress, defined as sustained nasal flaring, subcostal recessions; 4) multiple convulsions, defined as a respective history within the preceding 24 h plus one directly observed convulsion; 5) impaired consciousness, defined as a Blantyre score ≤ 4 [31]; 6) clinical jaundice; 7) circulatory collapse, defined as a systolic blood pressure < 60 and <80 mm of Hg in children ≤5 and >5 years of age respectively, plus cool limbs or weak or absent peripheral pulses; 8) abnormal bleeding; 9) pulmonary edema and 10) frequent vomiting [14]. The term cerebral malaria was reserved for Blantyre coma score ≤ 2 corrected for no record of recent severe head trauma, neurological disease, or other causes of febrile encephalopathy such as meningitis (assessed by the examination of cerebrospinal fluid) [32]. Uncomplicated malaria (UM) was defined as being fully conscious with haemoglobin ≥8 g/dl and no signs of severity and/or evidence of vital organ dysfunction [23].

The WHO scheme [14, 23] was used to further classify SM based on the degree of severity, into 3 groups with the first group being the most severe and associated with the highest mortality rate, the second group being moderately severe and the third being the least severe.

### Data analysis

Data collected were entered into an Excel spreadsheet and analysed using the Stata® version 12.1 software (StataCorp LP, Texas, USA) and the SPSS version 16 for Microsoft windows. The statistical tests performed included the Pearson’s Chi-square for comparison of proportions, the Student’s T-test and ANOVA for the comparison of group means, risk ratio (RR) for assessment of the risk for severe malaria, odd ratio (OR) for the identification of predictors of mortality and Multivariate regression analysis for the comparison of the geometric mean parasite density (GMPD) between groups adjusting for possible confounding. Statistical significance was set at *p* < 0.05.

## Results

### Characteristics of the study population

One thousand seven hundred and two (1702) participants were approached, 1609 met the inclusion criteria and were therefore enrolled; 318, 318, 341, 315, and 317 children were enrolled from Bamenda, Limbe, Maroua, Ngaoundere, and Yaounde respectively (Table 1). Among them were 779 (48.4%) females and 830 (51.6%) males. The ages of the participants ranged between 0 and 180 months (mean ± SD = 63.65 ± 56.69).

**Table 1:** Distribution of the study population with respect to age, gender and study site

Epidemiological strata	Study sites			Age (months)			Total
				< 60	60–119	120+	
HWP	Bamenda	Gender	F	70 (40.5)	29 (16.8)	74 (42.8)	173 (54.4)
			M	58 (40.0)	38 (26.2)	49 (33.8)	145 (45.6)
		Total		128 (40.3)	67 (21.1)	123 (38.7)	318
C	Limbe	Gender	F	75 (50.3)	39 (26.2)	35 (23.5)	149 (46.9)
			M	103 (61.0)	39 (23.1)	27 (15.9)	169 (53.1)
		Total		178 (56.0)	78 (24.5)	62 (19.5)	318
SCEF	Yaounde	Gender	F	61 (40.7)	32 (21.3)	57 (38.0)	150 (44.0)
			M	100 (52.4)	46 (24.1)	45 (23.6)	191 (56.0)
		Total		161 (47.2)	78 (22.9)	102 (29.9)	341
SS	Maroua	Gender	F	122 (74.4)	27 (16.5)	15 (9.1)	164 (52.1)
			M	111 (73.5)	27 (17.9)	13 (8.6)	151 (47.9)
		Total		233 (74.0)	54 (17.1)	28 (8.9)	315
HIP	Ngaoundere	Gender	F	94 (65.7)	32 (22.4)	17 (11.9)	143 (45.1)
			M	116 (66.7)	34 (19.5)	24 (13.8)	174 (54.9)
		Total		210 (66.3)	66 (20.8)	41 (12.9)	317
Total		Gender	F	422 (54.2)	159 (20.4)	198 (25.4)	779 (48.4)
			M	488 (58.8)	184 (22.2)	158 (19.0)	830 (51.6)
		Total		910 (56.6)	343 (21.3)	356 (22.1)	1609

*HWP* High western plateau strata, *C* Coastal strata, *SCEF* South Cameroonian Equatorial strata, *SS* Sudano-sahelian strata, *HIP* High inland plateau strata, *F* female, *M* male

Data are presented as number (%)

The mean (±SD) temperature, haemoglobin concentration, and blood glucose of the participants was 37.78 °C (±0.89), 9.6 g/dl (±1.3), and 6.6 mmol/l (±1.1) respectively.

### Distribution of malaria in the study population

Among the 1609 participants, 242 were positive for malaria parasites giving an overall prevalence of 15.0% (95% CI: 13.3–16.9). The prevalence of malaria was highest in Limbe (C strata) 27.4% (87/318; 95% CI: 22.5–32.6) followed by Yaounde (SCEF strata) 18.2% (62/341; 95% CI: 14.2–22.7), Bamenda (HWP strata) 14.5% (46/318; 95% CI: 10.8–18.8), Ngaoundere (HIP strata) 8.8% (28/317; 95% CI: 6.0–12.5), and lowest in Maroua (SS strata) 6.0% (19/315; 95% CI: 3.7–9.3). A significant association was observed between prevalence of malaria and study site (*p* < 0.001).

Overall no significant association was observed between the prevalence of malaria and gender (*p* = 0.481, Table 2).

**Table 2:** Distribution of malaria in the study population stratified according to age, gender and study site

Study site	Gender						Age category (months)							
	Female		Male		χ^2^	*P*-value	<60		60–119		120+		χ^2^	*p*-value
	n	Pos (%)	n	Pos (%)			n	Pos (%)	n	Pos (%)	n	Pos (%)		
Bamenda	173	28 (16.2)	145	18 (12.4)	0.907	0.341	128	12 (9.3)	67	15 (22.4)	123	19 (15.5)	6.175	0.045
Limbe	149	42 (28.2)	169	45 (26.6)	0.097	0.755	178	40 (22.5)	78	33 (42.3)	62	14 (22.6)	11.622	0.003
Yaounde	150	24 (16.0)	191	38 (19.9)	0.857	0.355	161	20 (12.4)	78	22 (28.2)	102	20 (19.6)	8.997	0.011
Maroua	164	11 (6.7)	151	8 (5.3)	0.275	0.600	232	14 (6.0)	54	3 (5.6)	28	2 (7.1)	0.083	0.959
Ngaoundere	143	13 (9.1)	174	15 (8.6)	0.022	0.833	210	17 (8.1)	66	7 (10.6)	41	4 (9.8)	0.644	0.725
Total	779	118 (15.2)	830	124 (14.9)	0.014	0.481	910	103 (11.3)	343	80 (23.3)	356	59 (16.6)	28.936	<0.001

With respect to age, the prevalence of malaria was highest in participants between 60 and 119 months and lowest in those below 60 months (Table 2). We found a significant association between the prevalence of malaria and age (*p* < 0.001).

**Fig. 2:**
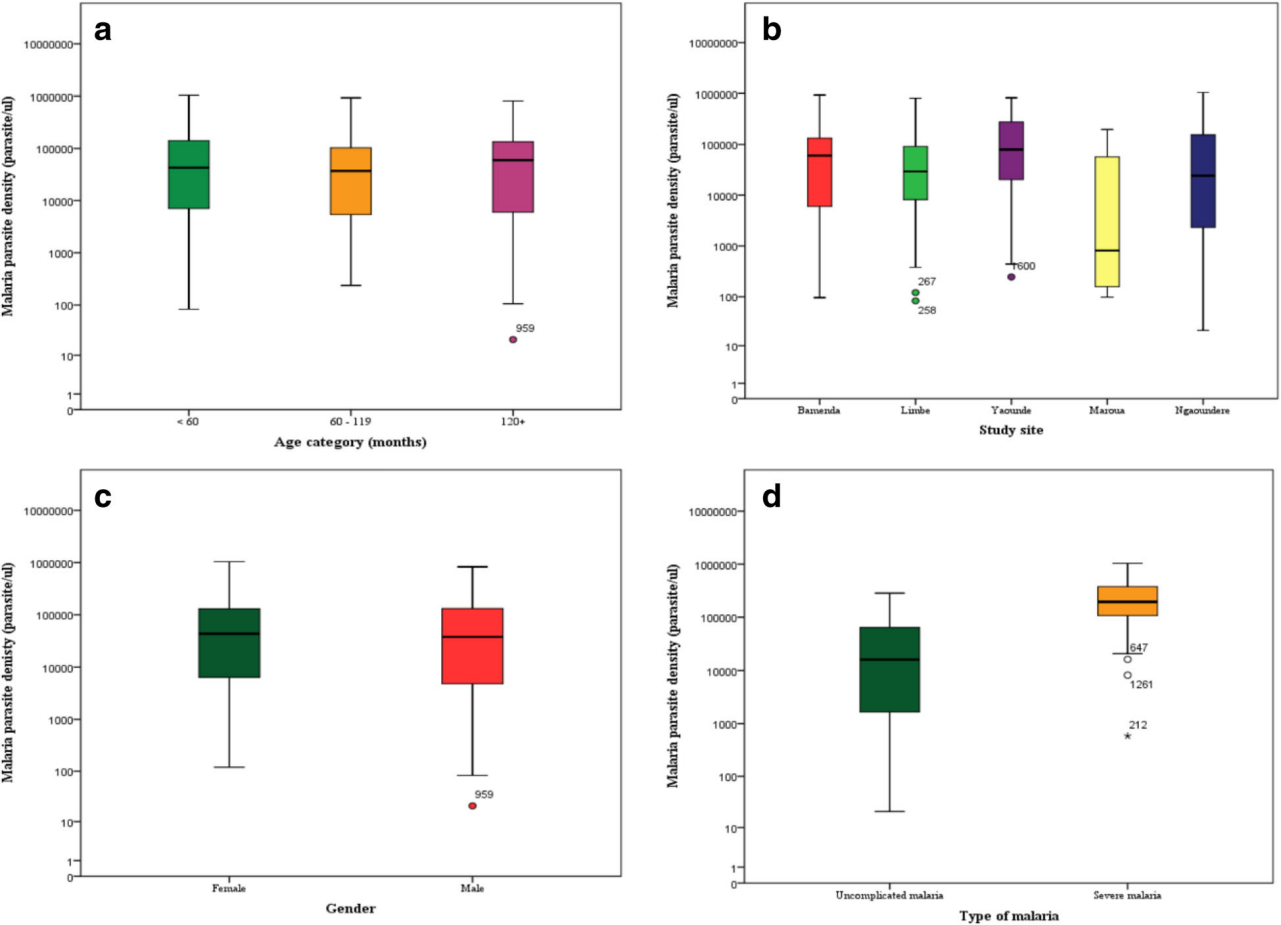
Boxplot of the malaria parasite density in the study population. Participant’s age was not observed to influence the parasite density (**a**) neither was the gender (**c**); but a significant difference was observed in the parasite density between the different study sites (**b**) and between uncomplicated and severe malaria (**d**)

Globally, the geometric mean parasite density (GMPD) was 22,576.9 parasites/μl (95% CI: 16,689.3–30,541.5). This was highest in children above 120 months of age, and lowest in children <60 months (Fig. 2a). Multivariate analysis revealed no significant difference in the parasite density in the different age groups adjusting for sex (*p* = 0.081). The GMPD was highest in Yaounde (SCEF strata) and lowest in Ngaoundere (HIP strata) (Fig. 2b). Adjusting for age and sex, a significant association was observed between parasite density and study site (*p* = 0.004). The GMPD was higher in females compared to males (Fig. 2c). However multivariate analysis revealed no significant difference in the parasite density between females and males adjusting for age (*p* = 0.353). The GMPD was significantly higher in SM cases compared to UM cases (Fig. 2d, *p* <0.001) adjusting for age and gender.

*Plasmodium falciparum* was identified in all (100%) the cases of malaria by microscopy. No mixed infection with the other *Plasmodium* species was identified.

### Distribution of severe malaria in the study population

Among the 242 malaria cases, 71 (29.3%) had severe malaria. The rate of SM attack was highest in children below 60 months (39.2%) (Table 3). A significant association was observed between the rate of SM attack and age (*p* = 0.009). The risk of SM was equally highest in children below 60 months (RR = 2.2, *p* = 0.003). The rate of SM attack was higher in males (31.5%) compared to females (27.1%). However, this difference was not significant (*p* = 0.459). The rate of SM attack was lowest in Limbe (22.1%) and highest in Maroua (42.1%) (Table 3). But, the association between SM attack and study site was not significant (*p* = 0.236). Moreover, the risk of SM attack was similar between the study sites (Table 3).

**Table 3:** The distribution of SM with respect to age, gender and study site

Parameter		UM	SM	Total	RR (95% CI)	*p*-value	χ^2^	*p*-value
Age	<60	64 (61.5)	40 (38.5)	104 (42.7)	2.2 (1.3–3.7)	0.003	9.326	0.009
	60–119	65 (82.3)	14 (17.7)	79 (33.1)	1.00			
	120+	42 (71.2)	17 (28.8)	59 (24.2)	1.6 (0.8–3.0)	0.150		
	Total	171 (70.7)	71 (29.3)	242				
Gender	Male	85 (68.6)	39 (31.5)	124 (51.5)	1.2 (0.8–1.7)	0.483	0.548	0.459
	Female	86 (72.9)	32 (27.1)	118 (48.5)				
	Total	171 (70.7)	71 (29.3)	242				
Study sites	Bamenda	33 (71.7)	13 (28.3)	46 (19.0)	1.3 (0.7–2.4)	0.523	5.540	0.236
	Limbe	67 (77.9)	19 (22.1)	86 (36.0)	1.00			
	Yaounde	39 (62.9)	23 (37.1)	62 (25.6)	1.7 (1.0–2.8)	0.064		
	Maroua	11 (57.9)	8 (42.1)	19 (7.9)	1.9 (1.0–3.7)	0.086		
	Ngaoundere	21 (72.4)	8 (27.6)	29 (12.4)	1.3 (0.6–2.5)	0.614		
	Total	171 (70.7)	71 (29.3)	242				

### Major clinical and prognostic features

1. **Cerebral malaria (CM)**

**Fig. 3:**
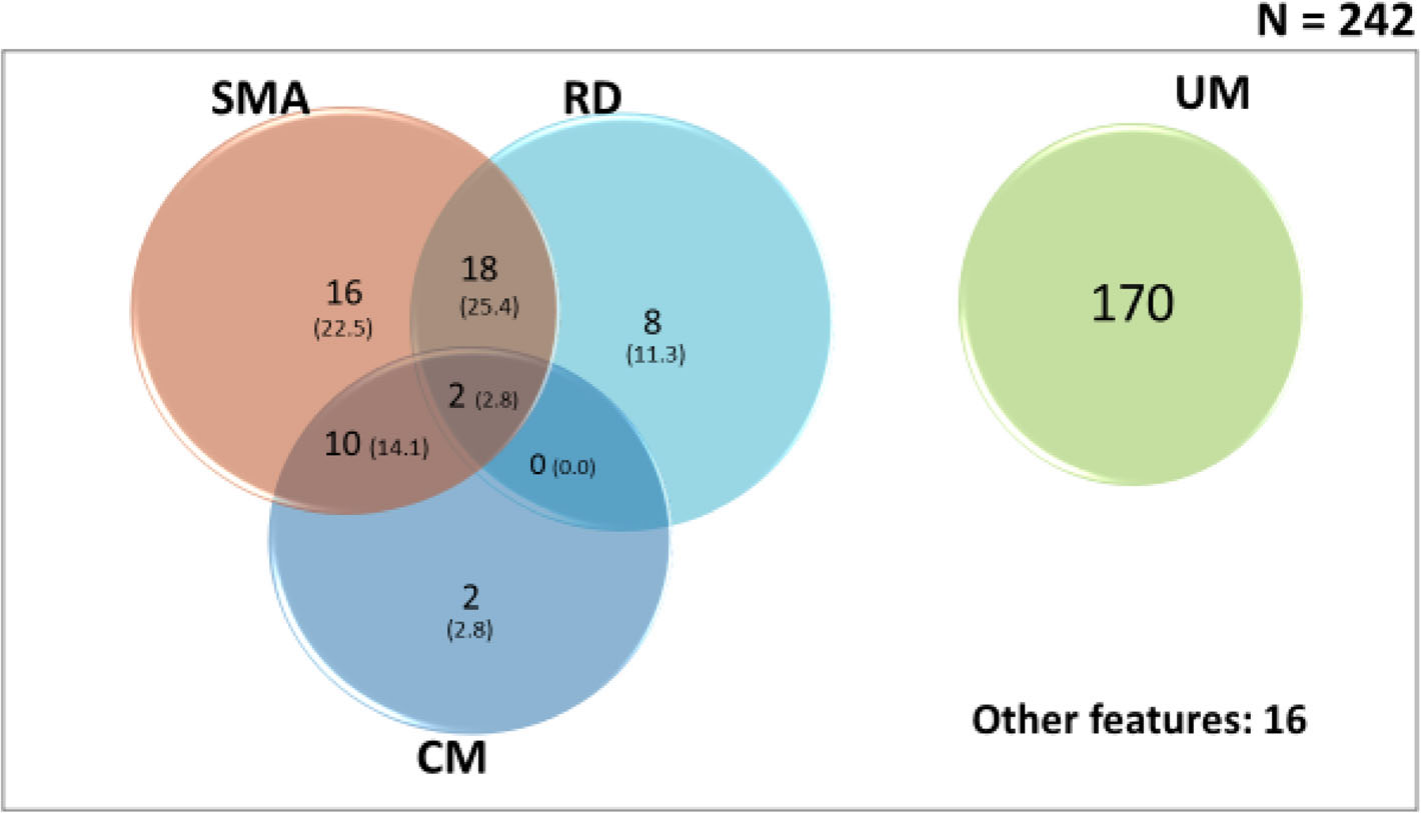
Venn diagram showing the overlap (proportions) of the major clinical subgroups of malaria in the study population. Proportions were obtained by dividing the cases by the total number of severe malaria (71). SMA: severe malarial anaemia; CM: cerebral malaria; RD: respiratory distress; UM: uncomplicated malaria

In all, 19.7% (14/71) of the participants had CM (Blantyre Coma Score ≤ 2). The proportion of CM was similar in all age groups (*p* = 0.910), and the proportion of males and females with CM was also similar [4.3% vs. 7.3%, *p* = 0.314] (Table 4). The frequency of CM was highest in Maroua (SS strata) and lowest in Limbe (C strata) (*p* = 0.003). A majority of children with CM had severe malaria anaemia (SMA) compared to those without CM (85.7% vs. 42.9%). Fewer children with CM had RD compared to those without (14.3% vs. 56.3%, Fig. 3). The mean haemoglobin, and blood glucose among patients with CM were 5.4 g/dl, and 5.9 mmol/l respectively (Table 5). The total case fatality ratio among children with CM was 28.6% (4/14) (Table 7).

2) **Severe malaria anaemia (SMA)**

**Table 4:** Distribution of the major phenotypes of severe malaria stratified according to age, gender and study site

Parameters	N	SMA	*p*-value	CM	*p*-value	RD	*p*-value	UM	*p*-value
Age category									
<60	103	28 (27.7)	0.046	7 (6.8)	0.910	17 (16.5)	0.048	63 (61.2)	0.007
60–119	80	11 (13.8)		4 (5.0)		4 (5.0)		66 (82.5)	
120+	59	9 (15.3)		3 (5.1)		6 (10.2)		42 (70.7)	
Gender									
F	118	21 (18.1)	0.639	5 (4.3)	0.314	15 (12.9)	0.454	86 (72.9)	0.459
M	124	25 (20.3)		9 (7.3)		12 (9.8)		85 (68.6)	
Study sites									
Bamenda	46	10 (21.7)	0.223	1 (2.2)	0.003	7 (15.2)	0.914	33 (71.7)	0.178
Limbe	86	12 (14.0)		1 (1.2)		9 (10.5)		68 (79.1)	
Yaounde	62	11 (17.7)		7 (11.3)		6 (9.7)		39 (62.9)	
Ngaoundere	29	6 (20.7)		1 (3.5)		3 (10.3)		20 (69.0)	
Maroua	19	7 (36.8)		4 (21.1)		2 (10.5)		11 (57.9)	

*SMA* severe malaria anaemia, *CM* cerebral malaria, *RD* respiratory distress, *UM* uncomplicated malaria

**Table 5:** Characteristics of the major clinical phenotypes

Parameters	CM (*n* = 14)	SMA (*n* = 46)	RD (*n* = 27)	*p*-value
Mean haemoglobin, g/dL (SD)	5.4 (1.6)	4.2 (1.8)	6.1 (2.2)	0.007
GMPD, parasites/μL	137,131.2	200,917.2	174,751.8	<0.001
Mean blood glucose, mmol/L (SD)	5.9 (0.8)	5.6 (1.3)	6.2 (1.1)	0.264
Mean body Temperature, °C (SD)	39.9 (0.9)	39.6 (1.1)	39.5 (1.1)	0.390
Hypoglycaemia, n (%)	7 (50.0)	15 (32.6)	9 (33.3)	0.471
Hyperparasitaemia, n (%)	9 (64.3)	16 (34.8)	6 (22.2)	0.028
Hyperpyrexia, n (%)	9 (64.3)	25 (54.4)	13 (48.2)	0.615

*CM* cerebral malaria, *SMA* severe malaria anaemia, *RD* respiratory distress, *GMPD* Geometric mean parasite density, *SD* standard deviation

This was the second most frequent manifestation 64.8% (46/710) that occurred in the affected children. SMA was more frequent in children <60 months (*p* = 0.046). The proportion of SMA was similar in males and females (20.3% vs. 18.1%, *p* = 0.639), and the proportion was also similar between the different study sites (*p* = 0.223) (Table 4). SMA had the highest GMPD (200,917.2 parasites/μl) (Table 5). The overlap of SMA with CM and RD were 14.1% (10/71) and 25.4% (18/71) respectively (Fig. 3). The total case fatality ratio among children with SMA was 26.1% (12/46) (Table 7).

3) **Respiratory distress (RD)**

About 38% (27/71) of the participants presented with RD at baseline. Children with RD were more likely to be younger and <60 months (*p* = 0.048). The proportion of RD was similar between males and females (9.8% vs. 12.9%, *p* = 0.454). The proportion was also observed to be similar between the different study sites (*p* = 0.914) (Table 4). RD was more common in SMA compared to CM [25.4% (20/71) vs. 2.8% (2/71)] (Fig. 3). The case fatality ratio of RD was 14.8% (4/27) (Table 7).

Other SM phenotypes at enrollment were hyperpyrexia 48 (67.6%), impaired consciousness 40 (56.3%), hypoglycemia 20 (28.2%), hyperparasitaemia 18 (25.4%), multiple convulsion 18 (25.4%), circulatory collapse 16 (22.5%), frequent vomiting 14 (19.7%), coma 14 (19.7%), jaundice 13 (18.3%), and prostration 12 (16.9%).

**Fig. 4:**
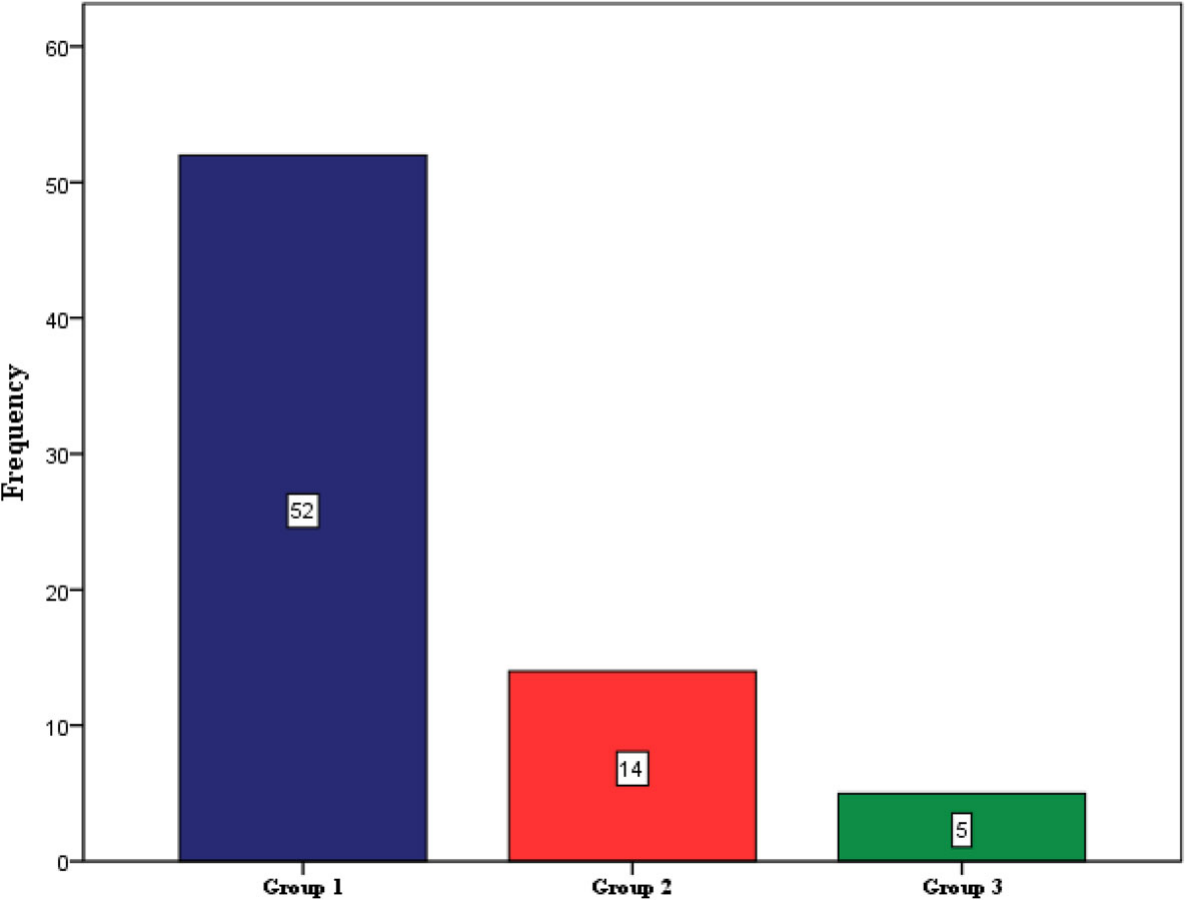
Distribution of SM cases according to the degree of severity. Group 1 is the most severe; group 2, moderately severe; and group 3, is the least severe form of SM

The majority of SM cases were in group 1 (i.e. most severe form of SM) on the WHO severity index, meanwhile group 3 (i.e. the least severe form of SM) had the least number of cases (Fig. 4).

Analysis of the degree of severity of SM revealed no significant association with gender (*p* = 0.616) or age (*p* = 0.377) or study site (*p* = 0.561) (Table 6).

**Table 6:** Distribution of the SM cases according to the degree of severity stratified by gender, age and study site

Characteristics	n	WHO classification of SM			Chi-square	*p*-value
		Group 1	Group 2	Group 3		
Gender						
M	39	28	9	2	0.970	0.616
F	32	24	5	3		
Age category						
< 60	40	31	8	1	4.221	0.377
60–119	14	10	3	1		
≥ 120	17	11	3	3		
Study sites						
Bamenda	13	9	3	1	6.773	0.561
Limbe	19	15	4	0		
Yaounde	23	18	2	3		
Maroua	8	5	2	1		
Ngaoundere	8	5	3	0		

### Factors associated with malaria death

The overall case fatality observed in this study was 5.8% (14/242). The case fatality rate were 7.8% (8/103), 3.7% (3/81), 5.1% (3/59) for age group <60, 60–119, ≥120 respectively. However no significant association was observed between fatality rate and age (*p* = 0.486).

The case fatality rate was higher in males 8.1% (10/123) compared to females 5.9% (7/119). However no significant association was observed between fatality rate and gender (*p* = 0.494).

The case fatality rate were 2.2% (1/46), 1.2% (1/86), 9.7% (6/62), 10.5% (2/19) and 13.8% (4/29) for HWP, C, SCEF, SS, and HIP strata respectively. A significant association was observed between the fatality rate and study site (*p* = 0.034).

Univariate analysis revealed that coma, hyperparasitaemia, hypoglycaemia, circulatory collapse, cerebral malaria, severe malarial anaemia, and jaundice were the risk factors associated with the most deaths (Table 7).

**Table 7:** Distribution of case fatality w.r.t the different prognostic indicators of malaria

Phenotype	n	Fatality (%)	Univariate analysis	
			OR (95% CI)	*p*-value
Impaired consciousness	40	3 (7.5)	1.00	
Hyperpyrexia	48	9 (18.8)	2.85 (0.72–11.34)	0.211
Respiratory distress	27	4 (14.8)	2.15 (0.44–10.46)	0.427
Jaundice	13	4 (30.8)	5.48 (1.04–28.97)	0.053
Severe malarial anaemia	46	12 (26.1)	4.35 (1.13–16.76)	0.044
Coma	14	12 (85.7)	74.0 (11.02–496.75)	<0.001
Convulsion	18	1 (5.5)	0.726 (0.07–7.49)	1.000
Circulatory collapse	16	6 (37.5)	7.4 (1.57–34.94)	0.012
Hyperparasitaemia	18	9 (50.0)	12.33 (2.76–55.05)	<0.001
Frequent vomiting	14	1 (7.1)	0.95 (0.09–9.95)	1.000
Prostration	12	1 (8.3)	1.12 (0.11–11.89)	1.000
Hypoglycaemia	20	9 (45.0)	10.09 (2.32–43.88)	0.001
Cerebral malaria	14	4 (28.6)	4.93 (0.95–25.74)	0.065
Uncomplicated malaria	168	3 (1.8)	0.22 (0.04–1.16)	0.087

## Discussion

The overall prevalence of malaria in the current study was 15.0%. This decreased steadily from the South towards the North of the country, being highest in Limbe (C strata) and lowest in Maroua (SS strata). The association between prevalence of malaria and study site was significant (*p* < 0.001). The variation in the prevalence of malaria between the study sites could be attributed to the different levels of transmission; malaria could be described as hyperendemic in Limbe [22], holoendemic in Yaounde [16, 33, 34], mesoendemic in Bamenda and Ngaoundere and hypoendemic in Maroua. Transmission of malaria in an area is known to be influenced by environmental variables including temperature, rainfall and humidity. Optimal temperature is needed for the development of the juvenile [35, 36], and adult stages of the mosquito vector [37], and optimal rainfall is equally needed for the creation of breeding sites. All the study sites surveyed offered favourable conditions for malaria transmission with the exception of Maroua which could be considered as arid with very little vegetation, low rainfall and very high temperatures in some months of the year. Thus, this may account for the low prevalence of malaria in the area. An entomological survey performed in Maroua also confirmed the low transmission of malaria in the area [38]. Site-specific analysis revealed a general decline in the prevalence of malaria in three of the study sites compared to earlier studies: the prevalence in Bamenda was lower compared to the 53.21% reported in 2006 [39], the prevalence in Yaounde was lower compared to the 34% reported in 2003 [40], and also lower in Ngaoundere compared to the 35% reported in 2006 [41]. The decrease in the prevalence of malaria in the current study could be accounted for by the increase in vector control chiefly by increased usage of ITNs which has been made available to almost every household in the country by the government [15, 16].

Malaria prevalence was higher in children between 60 and 119 months of age (*p* < 0.001), and this is consistent with studies performed elsewhere [42–44]; but contradicts the findings of other studies [45–47], in which no significant association was observed between prevalence of malaria and age. This trend was observed in all the sites except for Maroua, where the prevalence was highest in children aged 120 months and above. This could be attributed to the differences in the level of endemicity of malaria. In Maroua, malaria is hypoendemic and in hypoendemic areas, malaria is reported to be more common in older children compared to younger ones [48].

No significant association was observed between prevalence of malaria and gender in the current study (*p* = 0.459), which is consistent with studies performed elsewhere [27, 39, 42, 45–47]. Site-specific analysis also did not revealed any significant difference in the prevalence of malaria between males and females.

In the current study, *Plasmodium falciparum* was the only species identified as the cause of malaria. Molecular analysis further confirmed this [49]. The finding of *P. falciparum* as the sole cause of malaria in the target population is contrary to studies that have reported other *Plasmodium* species causing malaria including *P. vivax* [24, 50]. These discrepancies could be attributed to differences in the study designs; our study targeted febrile children presenting to hospitals in the different study sites meanwhile theirs targeted asymptomatic adults and children.

We found an overall geometric mean parasite density (GMPD) of 22,576.9 parasites/μl. This varied considerably across the different study sites, being highest in Yaounde and lowest in Maroua (*p* = 0.004). The observation of higher GMPD in Yaounde had previously been reported by Achidi et al. [22]. The precise reason for the underlying variation in malaria parasite density in different populations is not imminent. Some studies have shown that the parasite density is influenced by the time of specimen collection [51, 52], as well as uncontrolled environmental factors [53]. The GMPD was significantly higher in children with severe malaria (SM) compared to those with uncomplicated malaria (UM) (*p* < 0.001). High parasite density (hyperparasitaemia) is a known risk factor for severe malaria and this may explain the higher parasite density in severe malaria cases.

The rate of severe malaria (SM) attack in the current study was 29.3%. This rate is similar to that reported in a similar study [54], but differ considerable with those reported in other studies: the rate of SM attack was higher compared to the 12% reported in Sudan [4] and 19.4% reported in Nigeria [55]. The differences in the rate of SM in these studies and ours may be due to different levels of malaria endemicity in the study sites. The rate of SM attack in the current study was lower compared to the 61.8% reported in a study performed in Ghana [5]. This disparity could be attributed to the differences in the study design; our study targeted children of 15 years and below meanwhile the Ghanaian study targeted children of 59 months and below, a group that is most vulnerable to severe malaria attack [30]. The rate of SM attack was highest in children below 60 months in this study, which is in conformity with other studies [22, 23]. The high rate of severe malaria attack observed in children below 5 years of age is attributable to their low immunity, which has been observed to increase with age [41]. There was no significant association between rate of SM attack and gender, which is in conformity with studies performed elsewhere [23, 55]. The rate of SM although not significant, was observed to increase steadily from the South to the North of the country i.e. from Limbe in the Coastal strata (22.1%) to Maroua in the Sudano-sahelian strata (42.1%). It is important to note that this trend is in the opposite direction to that observed with the distribution of malaria. The increasing trend of SM attack towards the northern regions could be attributed to the differences in the exposure of the population residing in the different epidemiological strata, and this is related to their immunity against malaria parasites. The population in the C strata, where transmission is highest, are more exposed hence will tend to be more immune compared to the population in the other epidemiological strata. Studies designed to evaluate the immunologic responses to malaria parasites in the different epidemiological strata will therefore be required to confirm this hypothesis.

The main clinical phenotypes of SM in this study were hyperpyrexia, severe malaria anaemia, and impaired consciousness, which is in line with similar studies performed in other areas of Cameroon [22, 23, 56], and elsewhere [55]. Among the major clinical phenotypes, severe malaria anaemia (SMA) and respiratory distress (RD) were significantly higher in children below 60 months. The association between SMA and age in the current study is in conformity with studies by Achidi et al. [22]. The GMPD was also observed to be significantly higher in SMA cases suggesting that heavy parasitaemia may be important in the development of severe anaemia. On the other hand, cerebral malaria (CM) was significantly associated with the study site, with the prevalence being highest in Maroua (SS strata). The finding of a significant association between CM and study site is also in conformity with the study by Achidi et al. [22]. Contrary to the study by Achidi et al. [22], there was no association between SMA and gender or study site, and between RD and study site. The low number of severe malaria cases in our study may have accounted for these differences. In the current study, the majority of the SM cases were found in group 1 of the WHO classification of SM which corroborates the work of Kwenti et al. [23]. The distribution of SM in the different WHO categories was not associated with age, gender or the study site.

The overall case fatality rate was 5.8%, which is comparable to the rate reported by Achidi et al. [22]. The case fatality rate was however lower compared to the rate of 8.5% reported in Nigeria [55] and 14% reported in Uganda [54]. The case fatality rate reported in the current study may have been underestimated as some of the children might have died at home in the course of the study. The case fatality rate was not associated with gender nor age but did for the study sites (*p* = 0.034), increasing from Limbe (1.2%) northwards, where it peaked in Ngaoundere (13.8%). As mentioned above, this observation may be due to the variation in the immune responses which in turn is related to the differences in their exposure to the malaria parasites. Furthermore, univariate analysis revealed that coma, hyperparasitaemia, hypoglycaemia, circulatory collapse, cerebral malaria and severe malaria anaemia were all independent predictor of death.

This study which is one of the first of its kind, provides an interesting insight into the epidemiology of paediatric malaria in Cameroon, taking into consideration the variations across the different epidemiological strata of malaria. These findings may contribute significantly to programmes aimed at controlling malaria in the country. However, the seasonal variation of malaria and its clinical phenotypes were not investigated as the study was performed only during the rainy season during which malaria transmission is highest. These findings may not be generalizable to reflect the overall prevalence of malaria in the target population. Furthermore, study participants were recruited using health facilities in urban centers, these findings may not necessarily reflect the situation in rural areas. Larger studies involving rural areas will therefore be needed to shed more light.

## Conclusion

The overall malaria prevalence in this study was 15.0%. Malaria prevalence was significantly higher in Limbe in the Coastal strata and in children between 60 and 119 months. Malaria prevalence was observed to decrease steadily from the Coastal strata in the South to the Sudano-sahelian strata in the North, meanwhile the mortality rate associated with malaria increased in this direction. The rate of severe malaria attack was similar across the different study sites. Children below 60 months were most at risk of severe malaria attack. Hyperpyrexia, severe malaria anaemia and impaired consciousness were observed to be the main clinical presentation of severe malaria. The clinical phenotypes of severe malaria varied considerably across age groups and epidemiological strata; severe malaria anaemia and respiratory distress were more common in children below 60 months meanwhile cerebral malaria was significantly associated with the study site. Coma, hyperparasitaemia, hypoglycaemia, circulatory collapse, cerebral malaria and severe malaria anaemia were the independent predictor of mortality associated with malaria in the current study. Larger studies will therefore be required to shed more light on the seasonal variation of malaria and its clinical phenotypes in children in the different epidemiological strata.

## Abbreviation

C: Coastal strata
CM: Cerebral malaria
GMPD: Geometric mean parasite density
HIP: High inland plateau strata
HWP: High western plateau strata
RD: Respiratory distress
SCEF: South Cameroonian Equatorial strata
SM: Severe malaria
SMA: Severe malaria anaemia
SS: Sudano-sahelian strata
SSA: sub Saharan Africa
UM: Uncomplicated malaria
